# CD11c^+^ Cells Are Gatekeepers for Lymphocyte Trafficking to Infiltrated Islets During Type 1 Diabetes

**DOI:** 10.3389/fimmu.2019.00099

**Published:** 2019-01-31

**Authors:** Adam M. Sandor, Robin S. Lindsay, Nathan Dyjack, Jennifer C. Whitesell, Cydney Rios, Brenda J. Bradley, Kathryn Haskins, David V. Serreze, Aron M. Geurts, Yi-Guang Chen, Max A. Seibold, Jordan Jacobelli, Rachel S. Friedman

**Affiliations:** ^1^Department of Immunology and Microbiology, University of Colorado Anschutz Medical Campus, Aurora, CO, United States; ^2^Department of Biomedical Research, National Jewish Health, Denver, CO, United States; ^3^Center for Genes, Environment, and Health, National Jewish Health, Denver, CO, United States; ^4^The Jackson Laboratory, Bar Harbor, ME, United States; ^5^Department of Physiology, Medical College of Wisconsin, Milwaukee, WI, United States; ^6^Department of Pediatrics, Medical College of Wisconsin, Milwaukee, WI, United States; ^7^Department of Pediatrics, National Jewish Health, Denver, CO, United States; ^8^Division of Pulmonary Sciences and Critical Care Medicine, Department of Medicine, University of Colorado Anschutz Medical Campus, Aurora, CO, United States

**Keywords:** type 1 diabetes, CD11c^+^ cells, mononuclear phagocyte cells, T cell, lymphocyte trafficking, chemokine, extravasation

## Abstract

Type 1 diabetes (T1D) is a T cell mediated autoimmune disease that affects more than 19 million people with incidence increasing rapidly worldwide. For T cells to effectively drive T1D, they must first traffic to the islets and extravasate through the islet vasculature. Understanding the cues that lead to T cell entry into inflamed islets is important because diagnosed T1D patients already have established immune infiltration of their islets. Here we show that CD11c^+^ cells are a key mediator of T cell trafficking to infiltrated islets in non-obese diabetic (NOD) mice. Using intravital 2-photon islet imaging we show that T cell extravasation into the islets is an extended process, with T cells arresting in the islet vasculature in close proximity to perivascular CD11c^+^ cells. Antigen is not required for T cell trafficking to infiltrated islets, but T cell chemokine receptor signaling is necessary. Using RNAseq, we show that islet CD11c^+^ cells express over 20 different chemokines that bind chemokine receptors expressed on islet T cells. One highly expressed chemokine-receptor pair is CXCL16-CXCR6. However, NOD. CXCR6^−/−^ mice progressed normally to T1D and CXCR6 deficient T cells trafficked normally to the islets. Even with CXCR3 and CXCR6 dual deficiency, T cells trafficked to infiltrated islets. These data reinforce that chemokine receptor signaling is highly redundant for T cell trafficking to inflamed islets. Importantly, depletion of CD11c^+^ cells strongly inhibited T cell trafficking to infiltrated islets of NOD mice. We suggest that targeted depletion of CD11c^+^ cells associated with the islet vasculature may yield a therapeutic target to inhibit T cell trafficking to inflamed islets to prevent progression of T1D.

## Introduction

The recruitment of immune cells to sites of inflammation is one of the hallmarks of the immune response as well as a major therapeutic target in autoimmunity. Although blocking trafficking to the active site of disease has been shown to be efficacious in other autoimmune diseases, tools to inhibit recruitment of immune cells to inflamed islets, during type 1 diabetes (T1D) have not been successful. Cells within active sites of inflammation produce pro-inflammatory signals that lead to increased chemokines and integrin expression on inflamed vasculature. These changes to the inflamed vasculature then promote increased recruitment and entry of immune cells into these sites. (1, 2). While the process of leukocyte recruitment to inflamed tissues has been well-studied, many of the cellular and molecular signals that drive immune cell recruitment specifically to diabetic islets during the progression of T1D remain unclear.

T1D is characterized as a largely T cell mediated autoimmune destruction of the insulin producing β cells within the islets of Langerhans. This destruction of the β cells leads to dysregulation of blood glucose levels. Therapeutic options are limited to insulin replacement without treating the ongoing autoimmunity. Patients diagnosed with T1D maintain some beta cell mass despite having immune infiltration and destruction of many islets. Maintaining remaining beta cell mass is critical for preventing T1D progression and related complications. New lymphocytes are constantly being recruited to infiltrated islets (3). The incomplete understanding of how these lymphocytes traffic to the islets contributes to a lack of effective therapeutics to prevent immune cell recruitment in T1D progression.

In T1D, research (including our own) on the role of mononuclear phagocytes has focused on their role in antigen uptake and presentation to activate T cells (4–8). Notably, the majority of the mononuclear phagocytes in the islets express the marker CD11c (4). In T1D, islet CD11c^+^ cells are a mix of resident and recruited macrophages, dendritic cells (DCs), and inflammatory monocytes (4, 9, 10). Depletion of CD11c^+^ cells or removal of the pancreatic draining lymph node (PLN) prior to islet infiltration, prevents T1D progression in non-obese diabetic (NOD) mice (11–13). This suggests that the initial antigen priming of autoreactive T cells by CD11c^+^ cells occurs within the PLN. After islet infiltration, removal of the PLN in NOD mice no longer affects T1D disease progression, suggesting that further T cell recruitment and activation can occur within the pancreas (11). In experimental autoimmune encephalomyelitis (EAE), a mouse model of multiple sclerosis, CD11c^+^ cells are necessary for the recruitment of T cells to the inflamed CNS (14). However, in T1D, the role that CD11c^+^ cells play in the recruitment of T cells to inflamed islets remains unclear. These studies reinforce that there is a need to further understand the role of CD11c^+^ cells within the islets once infiltration has been established.

T cells that initially infiltrate the islets are thought to be islet antigen-specific (15). After initial infiltration, the requirement of antigen specificity for T cell trafficking to islets has yielded conflicting results (15–17). In a B10.BR.RIP-mHEL model of T1D, initial T cell infiltration is followed by up-regulation of chemokine and vascular adhesion molecule expression within the islets. These changes allowed for non-islet antigen-specific T cells to traffic to previously infiltrated islets (18). However, antigen was required for the accumulation of T cells in infiltrated islets of NOD retrogenic bone marrow chimera mice which expressed islet antigen-specific or non-specific TCRs (17). Due to these conflicting reports, the requirement of antigen for T cell trafficking to previously infiltrated islets remains unclear.

Chemokines are important for the directed recruitment of immune cells to sites of inflammation (1, 2). In T1D, more than half of all chemokines and chemokine receptors have been implicated in disease progression in both mouse and man (19). The chemokine superfamily is made up of more than 46 members in human, most of which have homologous members in mice. For immune cell trafficking to the islets, most studies focused on the role of CCL2, CCL3, CCL5, CXCL9, and CXCL10 (20–26). The chemokine CXCL16 has been reported as a potential candidate gene for the *Idd4* T1D risk locus in mouse (27), and its receptor, CXCR6, is located within IDDM22 T1D risk locus in man (28–30). Although it has been shown to have pathogenic properties in other autoimmune disease, the role of CXCL16 and CXCR6 have not been investigated in T1D.

We sought to identify the major requirements for T cells to traffic to the inflamed islets of NOD mice. Using intravital imaging, we show that T cell entry into the islets is an extended process, and intravascular T cells frequently arrest in close proximity to perivascular CD11c^+^ cells. We show that the presence of cognate antigen is not necessary for T cell trafficking to previously infiltrated islets, but T cell chemokine receptor signaling is required. Using RNA sequencing, we found that islet CD11c^+^ cells produce more than 20 chemokines that can recruit T cells to the islets. While CXCL16 is produced at high levels by islet CD11c^+^ cells, T cells deficient in its receptor CXCR6 can still traffic to infiltrated islets, even when combined with CXCR3 deficiency. However, depletion of CD11c^+^ cells profoundly impaired trafficking of lymphocytes to previously infiltrated islets. These data suggest that targeting CD11c^+^ cells within the islets may offer a therapeutic pathway to restrict T cell trafficking to previously infiltrated islets.

## Results

### T Cell Extravasation Into the Islets Is an Extended Process

Type 1 diabetes is caused by the T cell mediated destruction of the insulin-producing β cells within the islets. Before they can cause destruction of the β cells, T cells must enter the islets from the bloodstream (31). Previous work demonstrated that T cells within the islet vasculature arrested for prolonged periods of time, but T cell extravasation was not observed (32). Here, we analyzed T cell entry into the islets using an intravital pancreas imaging method that we developed (8). Islets were identified by vascular morphology and islet infiltration state was characterized as mild (0.1–30% of islet volume infiltrated by T cells) or advanced (30–60% of islet volume infiltrated by T cells) as previously described (8).

**Figure 1:**
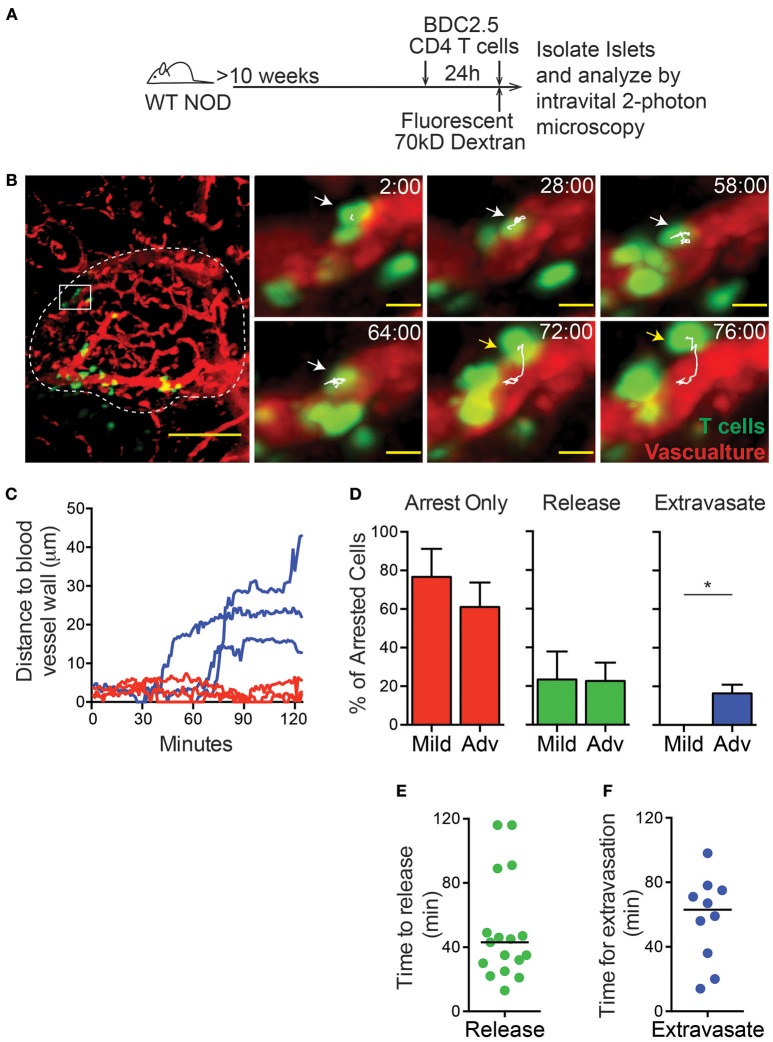
T cell extravasation into the islets is an extended process. Islet antigen-specific BDC-2.5 T cells were antigen-activated, fluorescently labeled, and transferred 24 h (to determine islet infiltration state, not shown) and immediately prior to imaging (to determine arrest and extravasation, green). Islets were imaged intravitally by 2-photon microscopy. **(A)** Schematic of experimental setup. **(B)** Representative islet image (dashed line) with T cells (green) and vascular volume (red). Scale bar = 50 μm. Right: T cell marked with arrow and track of motion is undergoing extravasation into the islet. Yellow arrow indicates completed extravasation. Time stamp = min:sec; Scale bar = 10 μm. **(C)** Each line represents the distance of the leading edge of one T cell from the surface of the blood vessel. Blue lines represent cells that completed extravasation; red lines represent arrested cells that did not complete extravasation. **(D)** Frequency of cells that remain arrested, release from arrest, or complete extravasation in mild and advanced infiltrated islets. **(E,F)** Dots indicate cells that arrested in the vasculature during the imaging period. Bar represents the median. **(E)** Time for T cell release from arrest in the islet vasculature. **(F)** Time to complete extravasation. **(D–F)** *n* = 6 islets from 5 mice in 5 experiments. **(D)** Error bars = SEM. *P < 0.05 calculated by Students *T*-test.

To identify the level of T cell infiltration in each islet, *in vitro* activated dye-labeled islet-antigen-specific BDC-2.5 CD4 T cells were intravenously (i.v.) transferred 24 h prior to imaging. Recipients were >10-week-old non-diabetic female NOD mice with established islet infiltration (Figure 1A). After 24 h, 90% of transferred BDC-2.5 CD4 T cells had extravasated into the islet parenchyma, while only 10% of transferred T cells remained in the islet vasculature (Figures S1A,B).

To analyze the process of T cell extravasation within the islets, additional *in vitro* activated differentially dye-labeled BDC-2.5 CD4 T cells were transferred immediately prior to intravital imaging (Figure 1A). Each islet was imaged by intravital 2-photon microscopy for 2 h. Individual T cells were tracked as they extravasated into the islets and moved away from the islet vasculature (Figures 1B,C; Video 1). Notably, we only observed the completion of extravasation through the microvasculature of the islet rather than islet-surrounding blood vessels (Figure 1B).

T cells that arrested in the vasculature were observed to complete the process of extravasation into the islets or more frequently release back into the blood flow (Figure 1D; Figure S2). Notably, completion of extravasation was only observed in advanced infiltrated islets, but not mild infiltrated islets within our 2-h imaging time. This indicates that the infiltration state within each islet can affect further T cell recruitment as extravasation becomes more permissive with increased infiltration (Figure 1D; Figure S2). Arrested T cells in the islet vasculature that released into the blood flow had a 43-min median time until their release, while completion of T cell extravasation had a median time of 63 min from the time of arrest (Figures 1E,F). Importantly, because many of the cells analyzed were arrested in the vasculature at the start or end of imaging, analyses of the duration of extravasation are underestimates. Extravasation into the islets was an extended process, more similar to extravasation into the highly restrictive CNS than to the permissive lymph nodes where T cell extravasation takes only 5–10 min (33–35). Therefore, the islet vasculature is highly restrictive to T cell extravasation.

### T Cells in the Islet Vasculature Are in Close Proximity to Perivascular CD11c^+^ Cells

**Figure 2:**
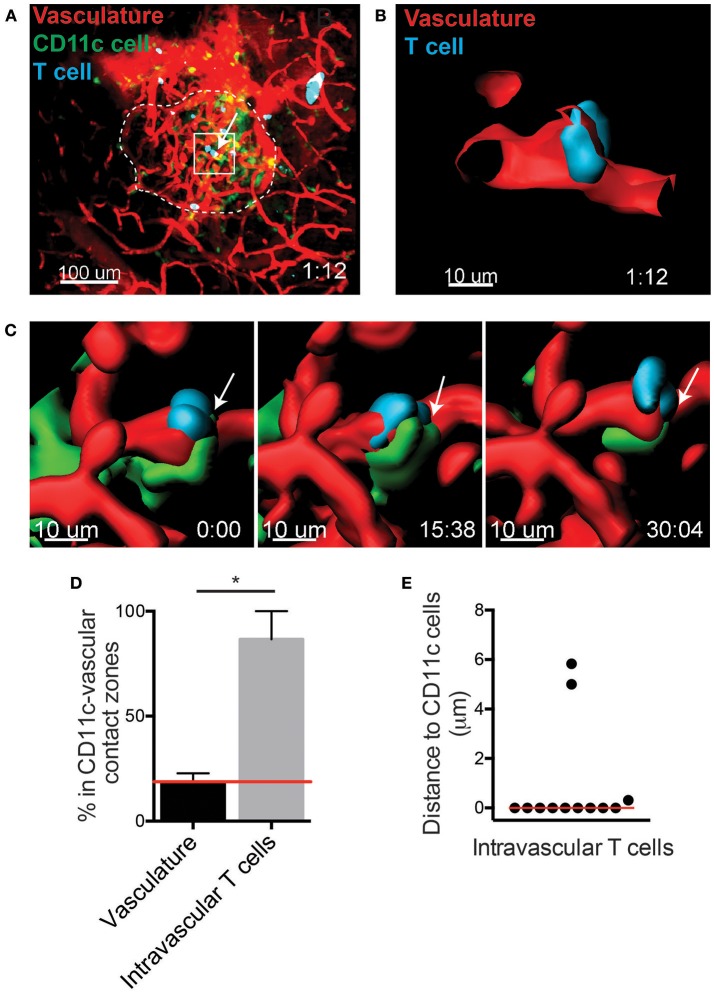
T cells arrest in close proximity to CD11c^+^ cells in the islet vasculature. Islet antigen-specific BDC-2.5 T cells (blue) were antigen-activated, fluorescently labeled, and transferred into NOD.CD11c-mCherry (green) mice. Islets were imaged intravitally by 2-photon microscopy. Vascular volume was labeled with fluorescent dextran (red). **(A)** Representative islet outlined by dashed line. Arrow indicates an intravascular T cell. **(B,C)** 3-Dimensional renderings created from the fluorescence in the boxed region in **(A)**. **(B)** Optical slice through the vascular lumen shows the T cell extending through the blood vessel wall. **(C)** Time lapse of T cell shown in **(B)**. Arrow indicates area of contact with perivascular CD11c^+^ cell (green). **(D)** Quantification of the percentage of vasculature area in contact with CD11c^+^ cells (CD11c-vascular contact zone) vs. the percentage intravascular T cells within CD11c-vascular contact zone. Red line indicates the predicted value for percentage of intravascular T cell within CD11c-vascular contact zones if T cell location within the vasculature was random. Error bars = SEM. *P < 0.05 calculated by Students *T*-test. **(E)** Analysis of the distance from intravascular T cells to the nearest CD11c^+^ cell. Bar = median. **(D,E)** *n* = 5 islets from 3 mice in 3 experiments.

The specific requirements for T cell entry into the islets remain unclear. A CD11c^+^ cell subset has been shown to be in contact with the islet vasculature (15). Thus, we asked whether CD11c^+^ cells contribute to the recruitment of T cells into the islets. To do so, islets were imaged using intravital 2-photon microscopy (Figure 2A). The distribution of T cells within the islet vasculature was identified using the imaging software Imaris (Figures 2A–C). Islet intravascular T cells and T cells undergoing extravasation (Figure 2B), were in close proximity to perivascular CD11c^+^ cells (Figure 2C; Video 2). To determine whether T cell arrest and extravasation in the vasculature was biased toward being in proximity to CD11c^+^ cells, we first quantified the regions of the islet vasculature that were in direct contact with CD11c^+^ cells (CD11c-vascular contact zones). The frequency of intravascular T cells that resided in or out of the CD11c-vascular contact zones was then quantified (Figures 2C–E). An average of 18.7% of the islet vasculature was in a CD11c-vascular contact zone. If the T cell distribution in the islets was random, we would expect that the frequency of intravascular T cells in CD11c-vascular contact zones to be 18.7%. Strikingly we found that 86.6% of the intravascular T cells were in CD11c-vascular contact zones (Figure 2D). Furthermore, 75.0% of intravascular T cells were in direct contact with CD11c^+^ cells (Figure 2E). This strong enrichment of intravascular T cells in CD11c-vascular contact zones suggests that CD11c^+^ cells may assist in T cell recruitment into the islets.

### Antigen Is Not Required for T Cell Trafficking to Previously Infiltrated Islets

CD11c^+^ cells are classically thought of as antigen-presenting cells, but the requirement for antigen in T cell trafficking to the islets has been controversial. It is thought that antigen is important for the initial wave of T cells to enter the islet as well as for the long-term accumulation of T cells in the islets (15, 17). Following islet infiltration, increased inflammation within the islets allows for non-islet antigen-specific T cells to traffic to the islets in a B10.BR.RIP-mHEL model of T1D (18). Furthermore, it has been shown that the majority of T cells that traffic to the islets of NOD mice have a naïve phenotype (3). To address if cognate antigen is a requirement for T cells to traffic to the islets of NOD mice we used the NOD.C6 mouse which has normal islet infiltration and disease progression, but lacks the antigen for the BDC-6.9 TCR transgenic T cells (36, 37).

**Figure 3:**
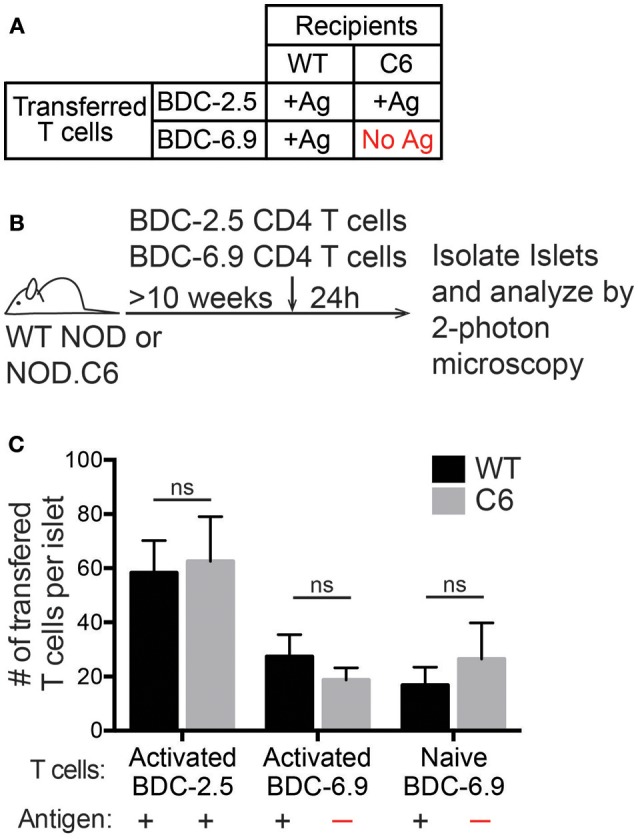
Antigen is not required for T cell entry into previously infiltrated islets. Islets in WT NOD mice contain the antigens for both BDC-2.5 and BDC-6.9 CD4 T cells. NOD.C6 mice lack the antigen for BDC-6.9 T cells but have the antigen for BDC-2.5 T cells and develop diabetes similar to WT NOD. BDC-2.5 or BDC-6.9 CD4 T cells were activated using αCD3 and αCD28, or naïve BDC-6.9 CD4 T cells were harvested from NOD.C6.BDC-6.9 mice. T cells were differentially fluorescent dye-labeled and co-transferred into NOD WT or C6 mice. Twenty-four hours after T cell transfer, islets were isolated and imaged using 2-photon microscopy to determine the number of transferred T cells that infiltrated the islets. **(A)** Table of transfer conditions. **(B)** Schematic of experimental setup. **(C)** Quantification of the number of islet BDC-2.5 T cells and islet BDC-6.9 T cells with or without antigen present. WT islets *n* = 25 from 4 experiments. C6 islets *n* = 18 from 5 experiments. All islets were previously infiltrated. Error bars = SEM. Statistical analysis by Students *T*-test.

The antigen for the BDC-2.5 CD4 T cell is present in both wild type (WT) NOD and NOD.C6 mice, while the antigen for the BDC-6.9 CD4 T cell is present in WT NOD but absent in NOD.C6 mice (Figure 3A). Thus, we tested if BDC-6.9 CD4 T cells could traffic to NOD.C6 islets in the absence of their cognate antigen (Figure 3). To do so, we co-transferred BDC-2.5 and BDC-6.9 CD4 T cells. Recipients were >10-week-old non-diabetic female WT NOD and NOD.C6 mice with established islet infiltration (Figure 3B). After 24 h, islets were harvested and the number of transferred T cells in the infiltrated islets was analyzed by 2-photon microscopy. The level of islet infiltration between WT NOD and NOD.C6 was similar as shown by similar numbers of BDC-2.5 CD4 T cells in WT NOD and NOD.C6 islets (Figure 3C). There was also no significant difference in the ability of both activated and naïve BDC-6.9 T cells to traffic to infiltrated islets regardless of whether their cognate antigen was present (WT NOD) or absent (NOD.C6) (Figure 3C). These data clearly show that antigen is not required for T cells to traffic to islets once infiltration has occurred.

### Chemokine Receptor Signaling Is Necessary for T Cell Trafficking to Previously Infiltrated Islets

Once initial infiltration occurs there are changes to the inflammatory state of the vasculature as well as increased chemokine production within the islets (15). Since antigen recognition is not required for T cell trafficking into the islets, we hypothesized that CD11c^+^ cells recruit T cells via chemokine production. To confirm that chemokine signaling is required for recruitment of T cells into previously infiltrated islets, we treated T cells with pertussis toxin (Ptx) to inhibit Gαi-coupled receptors, which include most chemokine receptors (38).

**Figure 4:**
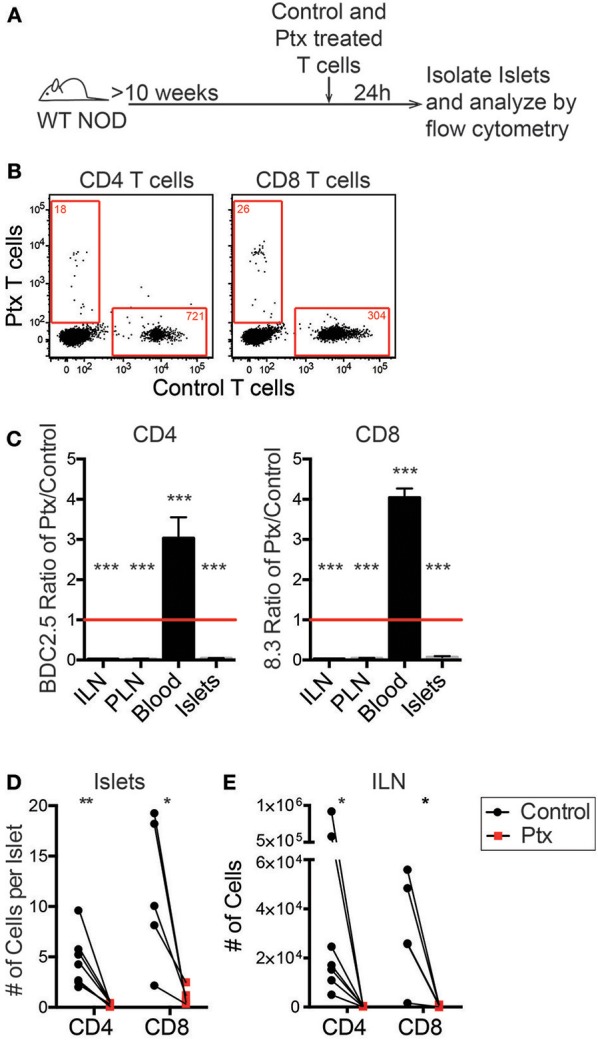
Chemokine receptor signaling is necessary for T cell trafficking to infiltrated islets. Antigen activated BDC2.5 (CD4) and 8.3 (CD8) T cells were treated with 200 ng of pertussis toxin (Ptx) or PBS for 2 h at 37°C and differentially dye-labeled. T cells were co-transferred into 10–16 weeks old female NOD mice. After 24 h ILN, PLN, blood, and pancreatic islets were isolated. The numbers of transferred T cells were determined by flow cytometry. **(A)** Schematic of experimental setup. **(B)** Representative flow plots of CD45+ cells comparing trafficking of WT and Ptx treated T cells to islets. Red numbers represent the number of cells in the gate. **(C)** Ratio of transferred Ptx treated to control T cells in each tissue analyzed. Statistics: One sample *T*-test with hypothetical value = 1. Error bars = SEM. **(D,E)** Quantification of the total number of transferred T cells that trafficked to **(D)** the islets and **(E)** the non-draining ILN. Statistics: Paired *T*-test. **(C-E)** *n* = 7 mice in 3 experiments for BDC2.5 CD4 T cells; *n* = 5 mice in 2 experiments for 8.3 CD8 T cells. *P < 0.05;**P < 0.01;***P < 0.001.

*In vitro* activated differentially dye-labeled BDC-2.5 CD4 and 8.3 CD8 T cells were pretreated for 2 h with 200 ng Ptx or vehicle control. Treated T cells were differentially dye-labeled, and then co-transferred into 10–16 week old WT NOD mice. After 24 h, the number of transferred cells was quantitated in the non-draining inguinal lymph node (ILN), islet-draining pancreatic lymph nodes (PLN), blood, and the islets by flow cytometry (Figure 4A). An average of 90% of T cells were localize to extravascular regions of islets at 24 h post-transfer (Figure S1). Ptx-treated T cells were significantly impaired in their ability to traffic to the islets and the lymph nodes (Figures 4B–E). This resulted in a low ratio of Ptx treated to vehicle treated CD4 and CD8 T cells in the ILN, PLN, and islets and an enriched ratio of Ptx to control treated T cells in the blood (Figure 4C). This suggests that the Ptx treated cells were viable but trapped in the blood. Ptx treatment of T cells led to a 95.6% impairment of CD4 trafficking and 91.3% of CD8 trafficking to the islets (Figure 4D) as well as a significant reduction in T cell entry into the non-draining ILN (Figure 4E). These data show that T cell chemokine receptor signaling is necessary for T cell trafficking to infiltrated islets.

### Islet CD11c^+^ Cells Express Many Chemokines That Pair With Chemokine Receptors on Islet T Cells

**Figure 5:**
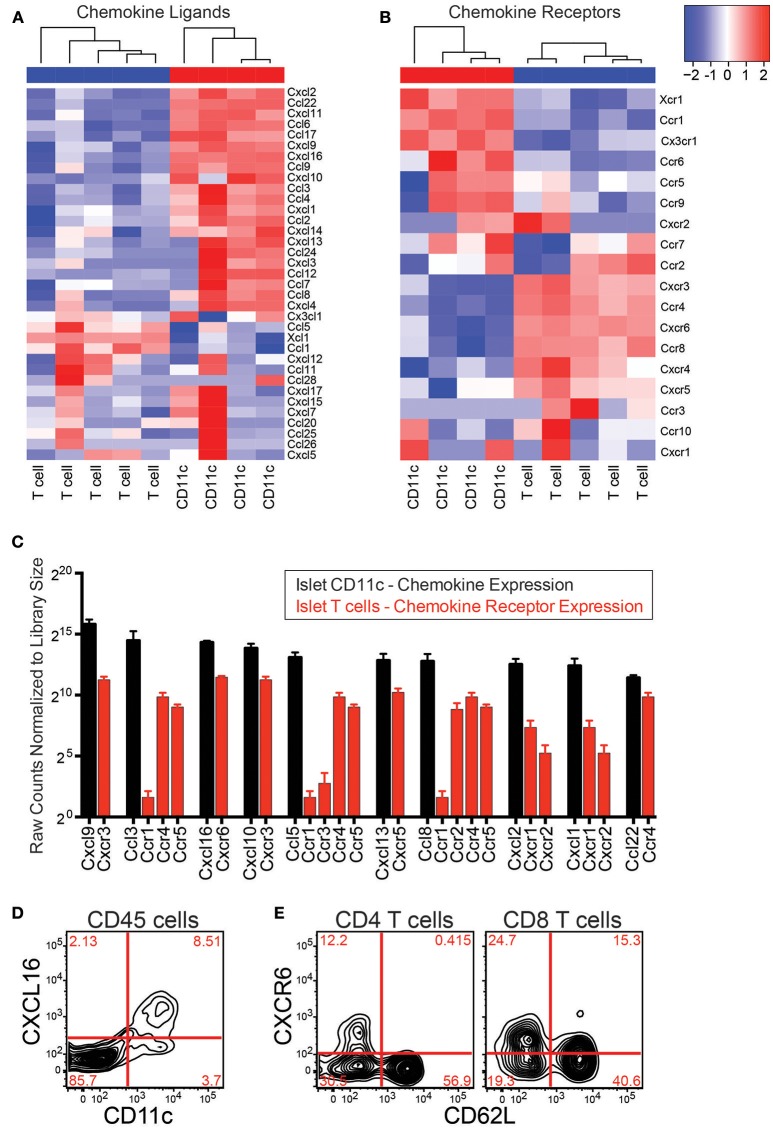
Islet CD11c^+^ cells express chemokines that pair with chemokine receptors on islet T cells. RNAseq was performed on CD11c^+^ cells (CD45^+^DAPI^−^CD19^−^CD11c^+^MHC-II^+^) and T cells (CD45^+^DAPI^−^CD19^−^CD90.2^+^) that were sorted from the islets of 12–20 wk NOD mice. Analysis of chemokine ligand and chemokine receptor expression in the islets was performed in R. **(A,B)** Heat map of normalized gene expression from islet CD11c^+^ cells and islet T cells. **(A)** Chemokine ligands. **(B)** Chemokine receptors. **(C)** Average expression of the top 10 expressed chemokine ligands by islet CD11c^+^ cells (black) and their receptor expression on islet T cells (red). Error bars = SEM. **(D)** Representative flow cytometry plot of CXCL16 expression on CD45^+^ cells in the islets. **(E)** Representative flow cytometry plots of CXCR6 expression by T cells in the islets.

If CD11c^+^ cells directly recruit T cells to the islets via chemokine production, they must produce chemokines that islet T cells can respond to. To determine if CD11c^+^ cells express chemokines that can bind islet T cell chemokine receptors, RNAseq was performed on islet CD11c^+^ cells and islet T cells. Islet CD11c^+^ cells (CD45^+^DAPI^−^CD19^−^CD11c^+^MHC-II^+^) and T cells (CD45^+^DAPI^−^CD19^−^CD90.2^+^) were FACS sorted and the RNA was isolated, amplified, and sequenced. RNA count data was normalized with DESeq2. Expression of chemokines and chemokine receptors on islet CD11c^+^ cells and T cells was analyzed using R (Figures 5A,B). Notably, the islet CD11c^+^ cells expressed over 20 different chemokines (Figure 5A). Of the top 10 chemokines produced by islet CD11c^+^ cells, islet T cells expressed high levels of one or more corresponding chemokine receptors for each chemokine (Figure 5C). Two chemokine ligand-receptor pairs that stood out were CXCR3-CXCL9/CXCL10 and CXCR6-CXCL16. CXCL9 was the highest expressed chemokine by CD11c^+^ cells and CXCR3 was the second highest expressed chemokine receptor on T cells (Figure 5C). CXCR6 was the highest expressed chemokine receptor on islet T cells and CXCL16 was the third highest CD11c^+^ cell produced chemokine (Figure 5C).

The CXCR3-CXCL9/CXCL10 axis has been well-studied in the progression of T1D, showing that CXCR3 is involved in initial recruitment of T cells to the islets, and is required for effective recruitment of regulatory T cells to NOD islets (21, 39–41). Thus, instead of pursuing the CXCR3-CXCL9/CXCL10 axis, we decided to focus on the CXCR6-CXCL16 receptor-ligand pair since CXCR6 and CXCL16 have not yet been well-investigated in T1D (41).

The CXCL16 gene is located within the *Idd4* T1D risk locus in mouse (27), and CXCR6 is located within the IDDM22 T1D disease locus in man, making this chemokine-receptor pair of strong potential interest (28). This pathway also interested us since CXCR6 has been shown to have a role for trafficking of pathogenic T cells in other animal models of autoimmunity such as EAE and colitis (42–44). CXCL16 has also been shown to be elevated in EAE and during rejection of a transplant (42, 45–47). Flow cytometry was used to confirm protein expression of CXCL16 by islet CD11c^+^ cells and CXCR6 on islet T cells (Figures 5 D,E). CD11c^+^ cells selectively expressed CXCL16 within the islet leukocyte population (Figure 5D). Using CD62L down regulation as a surrogate marker for T cell activation, only activated CD4 T cells expressed CXCR6 (Figure 5E), while subsets of CD62L positive and negative CD8 T cells expressed CXCR6. These data show that CD11c^+^ cells produce multiple chemokines that can bind receptors on islet T cells. We next sought to determine if CXCR6 expression was necessary for T cell infiltration into previously infiltrated islets.

### CXCR6 Is Not Required for T Cell Trafficking to Infiltrated NOD Islets

**Figure 6:**
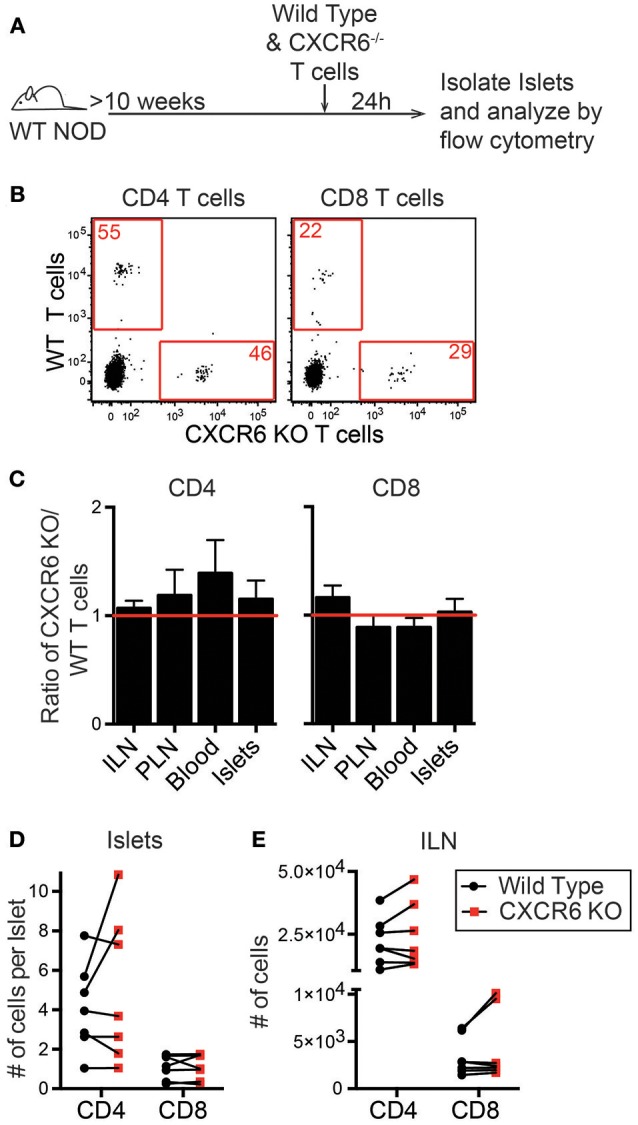
CXCR6^−/−^ T cells do not have impaired trafficking to NOD islets. NOD WT and CXCR6^−/−^ T cells were activated by αCD3 and αCD28, differentially dye-labeled, and co-transferred into 10–16 week old female NOD mice. After 24 h ILN, PLN, Blood, and pancreatic islets were isolated. Transferred T cells were quantified by flow cytometry. **(A)** Schematic of experimental setup. **(B)** Representative flow cytometry plots of CD45+ cells comparing trafficking of WT and CXCR6^−/−^ T cells to the islets. Red numbers represent the number of cells in the gate. **(C)** Ratio of transferred CXCR6^−/−^ to WT T cells in each tissue analyzed. Statistics: One sample *T*-test with hypothetical value = 1. **(D,E)** Number of transferred T cells that trafficked to **(D)** the islets normalized to the number of islets harvested and **(E)** to the non-draining ILN. Error bars = SEM. Statistics: Paired *T*-test. **(C-E)** *n* = 7 mice from 3 experiments.

To investigate if CXCL16 and CXCR6 have a role in T cell trafficking to previously infiltrated islets we used CXCR6^−/−^ NOD mice. Through PCR and flow cytometry we confirmed CXCR6 deficiency (Figures S3A,B). CXCR6^−/−^ NOD mice had no changes in T1D disease progression (Figure S3C). To test if CXCR6 deficiency impaired T cell trafficking to the islets, we differentially dye-labeled activated WT and CXCR6^−/−^ T cells and co-transferred them into 10–16-week old WT NOD mice. After 24 h ILN, PLN, blood, and islets were harvested, and the number of transferred cells was quantified by flow cytometry (Figures 6A,B). The ratio of CXCR6^−/−^ to WT T cells was compared within each tissue (Figure 6C). Trafficking of activated T cells was not impaired to any tissues analyzed, including the islets, as seen by an ~ 1:1 KO:WT ratio for transferred CD4 and CD8 T cells (Figure 6C). There was also no significant difference in the total number of CXCR6^−/−^ T cells compared to their co-transferred WT controls in either the islets (Figure 6D) or to the ILN (Figure 6E). CXCR6^−/−^ T cells still trafficked to the islets, likely due to the redundancy of chemokine and receptor pairs expressed within the islets (Figure 5C).

To determine if CXCR6 and CXCR3 dual deficiency could overcome the redundant usage of chemokine receptors in T cell trafficking to inflamed islets, we used the C57BL/6.RIP-mOVA model of T1D. C57BL/6.RIP-mOVA mice have membrane bound ovalbumin driven by the rat insulin promoter (48). In our colony, transfer of naïve OT-I CD8 T cells induces infiltration of the islets 6 days post-transfer without overt disease. We co-transferred differentially dye-labeled, activated C57BL/6.CXCR6^−/−^CXCR3^−/−^ double knock out and WT C57BL/6 T cells into C57BL/6.RIP-mOVA mice with pre-existing islet infiltration (Figure S4A). Surprisingly, the CXCR6^−/−^CXCR3^−/−^ T cells did not have a defect in trafficking to infiltrated islets (Figures S4B–E). These data show that deficiency in CXCR6 and CXCR3 is not sufficient to impair T cell trafficking to inflamed islets. There are over 20 chemokine and chemokine receptor pairs expressed by islet CD11c^+^ cells and T cells, respectively. This redundancy makes it infeasible to test all the combinations of CD11c-produced chemokines involved in T cell trafficking to infiltrated islets. Instead, we asked whether depletion of CD11c^+^ cells could disrupt T cell trafficking to the islets.

### Islet CD11c Depletion Is Effective and Does Not Affect T Cell Adhesion to the Islet Vasculature

**Figure 7:**
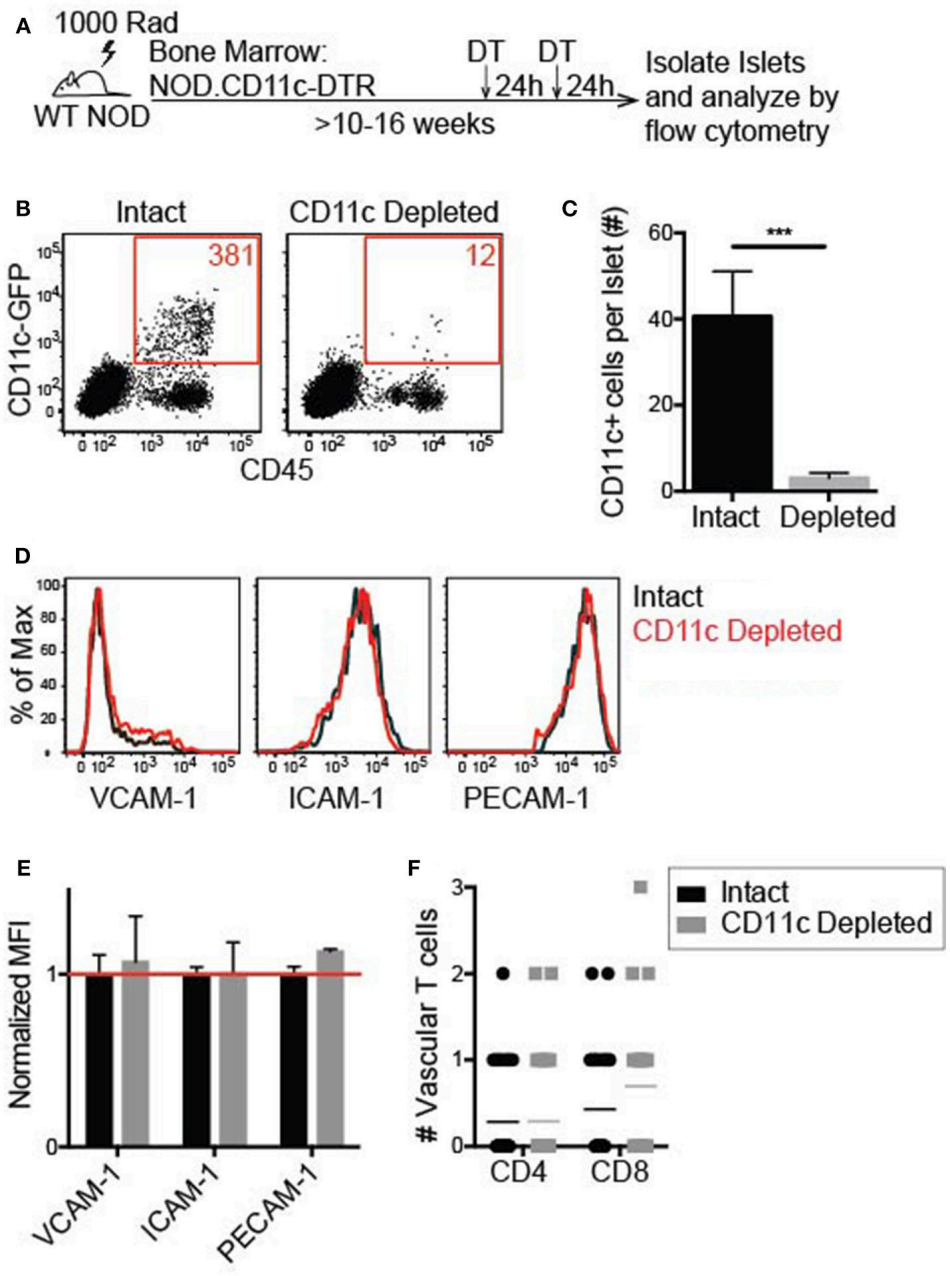
Islet CD11c^+^ cell depletion is effective and does not affect lymphocyte adhesion to the vasculature. Female NOD CD11c-DTR bone marrow was transferred into irradiated female NOD hosts to make bone marrow chimeras. 10–16 weeks post-reconstitution chimeras were treated twice 24 h apart with 200 ng of diphtheria toxin (DT) or PBS. Twenty-four hours after the second treatment, islets were isolated and digested. **(A)** Schematic for islet CD11c depletion. **(B,C)** Islet CD11c^+^ cell numbers were quantified by flow cytometry. **(B)** Representative flow cytometry plot of islet CD45+ cells. **(C)** Number of islet CD11c^+^ cells. Intact *n* = 12 mice, CD11c depleted *n* = 16 mice from 7 experiments; Error bars = SEM. Statistics: Students *T*-test, ***P < 0.001. **(D–G)** Flow cytometric analysis of adhesion molecule expression on endothelial cells (CD31+ CD45- cells) with or without CD11c depletion. **(D)** Representative histograms. **(E)** Adhesion molecule MFI normalized to the average MFI of intact islets. Red line signifies no change compared to control. *n* = 5 mice from 3 experiments. Statistics: One sample *T*-test with hypothetical value = 1. **(F)** Lymphocyte adhesion to the islet vasculature was analyzed by 2-photon whole islet imaging. Fluorescent dye-labeled, peptide-activated BDC-2.5 (CD4) and 8.3 (CD8) T cells were co-transferred 2 h prior to harvest. Islets were antibody stained for CD31 and T cell infiltration. *n* = 4 mice from 3 experiments. Statistics: Students *T*-test.

To effectively deplete CD11c^+^ cells in the islets, NOD.CD11c-DTR bone marrow (BM) chimeras were generated. CD11c-DTR mice express the high affinity diphtheria toxin receptor (DTR) under the CD11c promoter. Two treatments of diphtheria toxin (DT) 24 h apart led to a significant reduction in the number of CD11c^+^ cells within the islets 24 h after the final DT treatment (Figures 7A–C). A minimum of 90% CD11c depletion in the islets was required for inclusion in our analyses.

To determine whether vascular inflammation in the islets changed with CD11c depletion, the expression of the adhesion molecules ICAM-1 and VCAM-1 was quantified by flow cytometry (Figure 7D). PE-CAM-1 (CD31) was used to identify the vascular endothelial cells, but is also a vascular junction protein. Thus, the mean fluorescent intensity (MFI) of PE-CAM-1 was also quantified (Figure 7D). There was no difference in the MFI of ICAM-1, VCAM-1, or PE-CAM-1 when normalized to the average MFI of vehicle treated age-matched controls (Figure 7E). These data show that CD11c depletion does not alter adhesion molecule expression on the islet vasculature.

To determine if T cell adhesion to the islet vasculature was altered with CD11c depletion, activated T cells were dye-labeled and transferred 2 h prior to islet harvest. In whole islet preparations the vasculature was marked with αCD31 staining and T cells were marked genetically by CD2-dsRed or stained with αCD90.2 to identify the infiltration state of the islets. Islets were maintained in media with Ca^2+^ and Mg^2+^ during the harvest and stain. The number of transferred T cells within the vasculature of infiltrated islets was quantified by 2-photon microscopy. There was no significant difference in the average number of transferred T cells within the vasculature of intact vs. CD11c depleted islets (Figure 7F). These data suggest that CD11c^+^ cells in the islets can be depleted without altering the ability of T cells to adhere to the vasculature.

### Lymphocyte Entry Into the Islets Is Impaired Following CD11c Depletion

**Figure 8:**
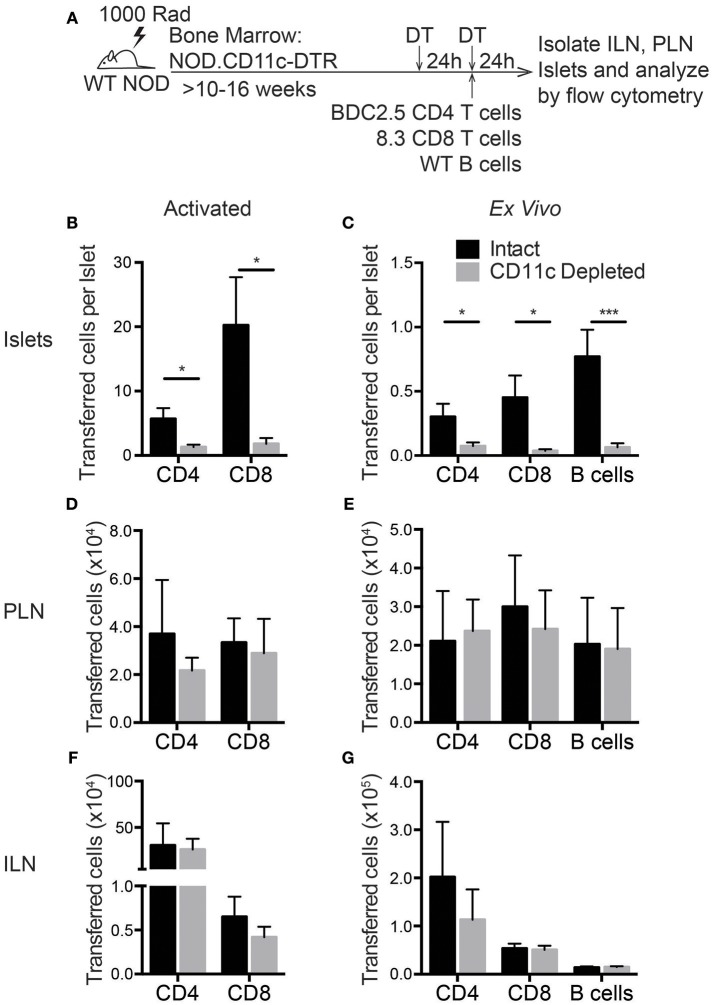
Lymphocyte entry into the islets is impaired by CD11c^+^ cell depletion. NOD.CD11c-DTR bone marrow chimera generation and CD11c depletion were done as described in Figure 7A. Negatively selected *ex vivo* or peptide-activated BDC-2.5 CD4+ and 8.3 CD8+ islet antigen-specific T cells and *ex vivo* B cells were fluorescent dye-labeled and co-transferred at the time of the second DT treatment. Twenty-four hours after cell transfer, ILNs and islets were isolated and digested. Transferred cells within the tissues were quantified by flow cytometry. **(A)** Schematic of experimental setup. **(B,C)** The number of **(A)** activated and **(B)** *ex vivo* transferred cells in the islets normalized to the total number of islets isolated. For *ex vivo*: *n* = 10–16 mice from 4–8 experiments. For activated: *n* = 6–8 mice from 3 experiments. **(D,E)** Number of **(C)** activated and **(D)** *ex vivo* transferred cells in the PLN. **(F,G)** Number of **(C)** activated and **(D)** *ex vivo* transferred cells in the ILN. **(D–G)** For *ex vivo*: *n* = 4–6 mice from 2 experiments. For activated: *n* = 6 mice from 3 experiments. Error bars = SEM. Statistics: Students *T*-test; *P < 0.05; ***P < 0.001.

Based on the RNAseq data, islet CD11c^+^ cells produce more than 20 different chemokines that could recruit lymphocytes to the islets (Figure 5C). To determine if these CD11c^+^ cells might have a required role in lymphocyte entry to infiltrated islets, we assessed trafficking of T cells to the islets after CD11c^+^ cell depletion. *In vitro* activated T cell trafficking to the islets was assessed to mirror the experimental setup of our extravasation and chemokine experiments. Trafficking of directly isolated NOD *ex vivo* T cells and B cells was also assessed to ensure the physiological relevance of the *in vitro* activated cells. *In vitro* activated or directly *ex vivo* isolated islet antigen-specific BDC-2.5 CD4 and 8.3 CD8 T cells as well as *ex vivo* B cells were differentially dye-labeled and transferred into vehicle (intact) or DT (CD11c Depleted) treated NOD.CD11c-DTR BM chimeras. After 24 h, the numbers of transferred cells in the non-draining ILN, draining PLN, and islets were quantified by flow cytometry (Figure 8A). Strikingly, short-term depletion of CD11c^+^ cells strongly impaired the numbers of T cells and B cells that were able traffic to the islets (Figures 8B,C). This impairment in trafficking was seen for both *in vitro* activated (Figure 8B) and *ex vivo* (Figure 8C) transferred T cells. However, lymphocyte trafficking to the PLN and ILN were not affected by CD11c depletion (Figures 8D–G). The impairment of T cell trafficking to the islets with CD11c depletion was profound, with CD8 T cells having a stronger impairment (92 and 91% reduction for *ex vivo* and activated) than CD4 T cells (75 and 77% reduction for *ex vivo* and activated). Interestingly, there was also a 92% impairment of B cell trafficking to the islets following CD11c depletion (Figure 8C). B cells also play a pathogenic role in T1D progression and can be recruited to the islets through chemokine signaling (49–53). These data show that CD11c^+^ cells are required for effective recruitment of lymphocytes into islets.

## Discussion

In this study, we sought to better understand the cellular and molecular cues that enable lymphocytes to traffic to diabetic islets. Our data show that CD11c^+^ cells are necessary for effective entry of lymphocytes into previously infiltrated NOD islets. We show that T cell extravasation into the islets is an extended process, indicating that the islet vascular barrier is highly restrictive to T cell entry. Notably, T cells in the islet vasculature were located proximal to CD11c^+^ cells, and required chemokine receptor signaling rather than antigenic stimulation to traffic into infiltrated islets. The fact that over 20 chemokine-receptor pairs are expressed by islet CD11c^+^ cells and islet T cells, respectively, highlights the large degree of redundancy for chemotactic cues in infiltrated islets. This redundancy enables T cells to traffic to the islets in the absence of individual chemokine receptors. The profound impairment in lymphocyte trafficking to inflamed islets in the absence of CD11c^+^ cells indicates that CD11c^+^ cells have a gatekeeper role for lymphocyte trafficking and entry into the islets, likely through production of multiple chemokines.

This profound dependence on CD11c^+^ cells for lymphocyte trafficking to the islets is highly significant in that the impairment is shown in NOD mice with established islet inflammation. Studies of T cell trafficking to the islets have largely analyzed trafficking to uninfiltrated islets (40, 54–57). Although these studies are important for protecting β cell mass in uninflamed islets, patients diagnosed with T1D already have established islet inflammation. Notably, many factors involved in T1D during disease induction, such as INFγ or CXCR3, become redundant once robust inflammation occurs within the islets (39, 58). In the context of inflamed islets, multiple redundant pathways must be blocked to prevent T cell entry into the islets as effectively as CD11c^+^ cell depletion. Examples of these include the combined blockade of vascular adhesion molecules or blockade of all chemokine receptor mediated signaling as we show with pertussis toxin (18, 59). This suggests that CD11c^+^ cells are likely key mediators of redundant pathways required for T cells to traffic to inflamed islets.

The ability of cells to traffic to the islets despite the absence of cognate antigen is intriguing and in agreement with previous reports (3, 15, 60). It has been shown that the majority of T cells trafficking to infiltrated islets in NOD mice have a naïve phenotype (3). Once naïve islet-antigen specific T cells traffic to the islets, they may become activated through antigen presentation by CD11c^+^ cells, since we have shown evidence of active antigen presentation by CD11c+ cells in the islets (7, 8). Non-islet antigen specific T cells have also been shown to traffic to the islets in inducible systems of T1D once islet inflammation is established (15, 60). Interestingly, the accumulation of non-islet antigen specific CD8 T cells is correlated with increased suppression of islet-antigen specific CD8 T cells (60). Therefore, although these non-islet antigen specific T cells are not causing increased β cell destruction, they could still have an effect on T1D progression.

We show that as with T cell trafficking, CD11c^+^ cells are required for effective B cell trafficking to the islets. This is likely due to the broad expression of chemokines produced by CD11c^+^ cells, since recruitment of B cells to the islets can be driven by chemokines (53). Although B cells play a pathogenic role in T1D and their depletion can slow disease progression in T1D patients, few studies have looked at the specific requirements for B cell trafficking to the islets (49–52, 61, 62). The combined inhibition of T cell and B cell trafficking to the islets may have greater potential to prevent T1D disease progression than blocking T cell trafficking alone.

CD11c^+^ cells in the islets include macrophages, dendritic cells, and monocytes that serve multiple roles during the progression of T1D (4, 7–10). Depletion of CD11c^+^ cells or removal of the draining PLN prior to islet infiltration prevents T1D, likely due to the lack of T cell priming by CD11c^+^ cells in the PLN (11–13, 57). However, here we show that islet CD11c^+^ cells have a previously undescribed role in the recruitment of lymphocytes to infiltrated islets. CD11c^+^ cells also enable recruitment of T cells through the restrictive blood brain barrier during CNS trafficking in EAE (14). This raises the question of whether CD11c^+^ cells are generally required for T cell trafficking to sites of inflammation, or if they are specifically required for T cell extravasation at restrictive vascular sites.

The fact that both CD11c^+^ cell depletion and Ptx-mediated chemokine inhibition led to ~90% inhibition of CD8 T cell trafficking to the islets suggests that CD8 T cells may be strongly reliant on CD11c^+^ cell produced chemokines for islet trafficking. This is surprising since other immune subsets and beta cells can produce a large array of chemokines in infiltrated islets (21, 63). Importantly, CD8 T cells are considered one of the main effector cells that lead to β cell death. On the other hand, since only 75% of CD4 T cell trafficking was inhibited by CD11c depletion, it is likely that CD4 T cells can respond to chemokines produced by other cell types in the islets.

Chemokines not only act as chemoattractants for lymphocytes, but they are also required for multiple steps of extravasation (1, 2). Soluble chemokines bind to the surface of CD11c^+^ cells and the vascular endothelium. These surface- bound chemokines on CD11c^+^ cells, as well as transmembrane chemokines such as CXCL16, can drive interactions of CD11c^+^ cells with T cells (1, 64–70). Here we show that not only are T cells in close proximity to perivascular CD11c^+^ cells, but many are in direct contact with CD11c^+^ cells. Intravascular T cells can interact directly with CD11c^+^ cells due to the ability of CD11c^+^ cells to periscope dendrites into the vascular lumen of the islets (15). Since antigen is not necessary for T cell trafficking to infiltrated islets, these interactions are likely driven by chemokines or integrin ligands presented to T cells by the CD11c^+^ cells (64–70). Notably, chemokine signaling can potentiate integrin affinity maturation. Both chemokine and integrin signaling drive cytoskeletal re-arrangements in T cells that are required for extravasation through endothelial barriers (71, 72). By increasing the local density of chemokines at endothelial junctions or on the extravascular basal membrane, CD11c^+^ cells may be responsible for enabling T cell path finding to permissive sites of extravasation (73–76).

During T1D, islet CD11c^+^ cells also produce cytokines such as TNFα, IL-1β, and VEGF that can increase vascular permeability (9, 63, 77, 78). Additionally, matrix metalloproteases (MMPs) are important for immune cell trafficking to the CNS during inflammation and to the islets during T1D (79–82). Through pro-inflammatory cytokine and MMP production, CD11c^+^ cells could break down vascular junctions and the basement membrane, creating permissive sites for T cells to transit through the vasculature. CD11c^+^ cell chemokine production would then attract T cells to the permissive vasculature created at the CD11c-vascular contact zones.

To more effectively identify a novel therapeutic target, further work must be done to understand the populations of CD11c^+^ cells in the islets that drive T cell recruitment. Although CD11c^+^ cells in the islet are a mixture of DC, macrophage, and monocyte populations, one specific subtype may be selectively required for T cell entry into the islets. Perivascular CD11c^+^ cells are likely responsible for T cell trafficking into the islets. Understanding the unique characteristics and markers of this islet CD11c^+^ cell subset may allow us to specifically target these cells to disrupt trafficking to the inflamed islets, without affecting normal T cell activation and immune function. This study highlights the additional work that needs to be done to understand the complexity of CD11c^+^ cells in the islets and their roles during the progression of T1D. However, we propose that targeting CD11c^+^ cell subtypes to alter their presence or function could provide a therapeutic target for broad inhibition of lymphocyte trafficking to the islets during T1D.

## Materials and Methods

### Mice

WT NOD (001976), NOD.8.3 (005868), and C57BL/6.RIP-mOVA (005431) were purchased from The Jackson Laboratory. C57BL/6.CXCR3^−/−^ (005796) and C57BL/6.CXCR6^−/−^ (005693) were also purchased from The Jackson Laboratory and crossed to generate C57BL/6.CXCR3^−/−^CXCR6^−/−^ dKO mice. The lab of Dr. Kathryn Haskins provided the NOD.BDC2.5, NOD.BDC6.9, NOD.C6, and NOD.C6.BDC-6.9 mice. The lab of Dr. Jonathan Katz provided the NOD.CD11c-DTR mice. Dr. Qizhi Tang provided the NOD.CD2-dsRed mice. NOD.CXCR6^−/−^ were generated by Dr. David Serreze at The Jackson Laboratory as previously described by CRISPR/Cas9 targeting of exon 2 of CXCR6 in NOD mice using the guide sequence CTCTTGATGCCCATCATCCA, resulting in a 7 base pair deletion (83). NOD.CXCR6^−/−^ were provided by Dr. Yi-Guang Chen, and are now available from The Jackson Laboratory (033094). PCR screening for the NOD.CXCR6^−/−^ was done by PCR using the following primers: forward-AGATGCCATGGATGATGG for which binding is disrupted by the 7bp deletion and reverse-CCAAAAGGGCAGAGTACA (Figure S3). The Institutional Animal Care and Use Committee at National Jewish Health approved all the procedures.

### Islet and Lymph Node Isolation and Digestion

Islets were isolated as previously described (7, 8). Briefly, mice were euthanized by i.p. administration of ketamine (50 μg/g) (Vedco)/xylazine (5 μg/g) (JHP) and cervical dislocation. The pancreas was inflated with 0.8% Collagenase P (Roche) and 10 μg/mL DNAse (Roche) in HBSS (Cellgro). Each lot of Collagense P was titrated for time necessary for digestion at 37°C between 11 and 14 min. Digested islets were separated by density centrifugation and hand picked under a dissection microscope. Pancreatic draining and inguinal lymph nodes we harvested and teased apart using syringe needles. For single cell suspension for flow cytometric analysis, lymph nodes and islets were digested for 30 min with 4 Wunsch units of Collagenase D (Roche) with 250 μg/ml DNAse in HBSS with 10% FBS. Islets were then incubated for 30 min in Cell Dissociation Buffer (Sigma).

### T Cell Isolation and *in vitro* Activation of T Cells

Lymphocytes were isolated from pooled lymph nodes and spleen cells. *Ex vivo* cells were harvested into EasySep buffer and CD4 T cells, CD8 T cells, and B cells were negatively selected using EasySep negative selection isolation kits (StemCell Technologies). For *in vitro* T cell activation, lymph node and spleen cells were *in vitro* activated using 24 well-plates coated with 2 μg/ml αCD3 (BioXcell) antibody and soluble 2 μg/ml αCD28 (BioXcell). Peptide activation of T cells was used for the BDC-2.5 and 8.3 TCR transgenics using the BDC-2.5 mimetope (YVRPLWVRME) (Pi Proteomics) or 8.3 cognate peptide (KYNKANVEL) (Chi Scientific). Beginning on day 2 post-stimulation, activated T cells were cultured with 10 IU/ml rhIL-2 (AIDS Research and Reference Reagent Program, Division of AIDS, NIAID, NIH from Dr. Maurice Gately, Hoffmann - La Roche Inc.). Cells were used 6–9 days post-initial T cell activation.

### Pertussis Toxin Treatment, Dye Labeling, and Adoptive Transfer of Lymphocytes

Activated T cells were resuspended at 10^7^ cells/ml and treated with 200 ng/ml Ptx (Hooke Labs) for 2 h at 37°C or with PBS as a vehicle control. For vital dye labeling, lymphocytes were resuspended at 10^7^ cells/ml and labeled using 1 μM VPD (BD), 2 μM CFSE (Invitrogen), 20 μM CMTMR (Invitrogen) or 5 μM eFluor 670 (eBiosciences). Lymphocytes were dyed at 37°C for 10 min for analysis by flow cytometry and for 25 min for microscopy. Dyes were switched between experiments. 10^7^ dye-labeled lymphocytes were adoptively transferred by i.v. injection into recipient mice.

### RNA Transcriptome Gene Expression and Quality Control

Sample RNA was isolated using the Quick-RNA Microprep kit (Zymo Research) according to the manufacturer's protocol. RNA AmpliSeq libraries were constructed and barcoded with the Ion AmpliSeq Transcriptome Mouse Gene Expression Kit and methods. Average RNA yields were 0.79 ± 0.95 ng. Because yields were <10 ng used as standard input for AmpliSeq, we instead loaded the maximum volume of RNA that the reaction could accommodate for all samples. Average inputs for Amplification were 0.64 ± 0.49 ng. Barcoded RNA sequencing (RNA-Seq) libraries were pooled and sequenced together on the Ion Torrent S5 sequencer by using P1 chips. Sequencing reads were mapped to AmpliSeq transcriptome target regions with the torrent mapping alignment program and quantified with the Ion Torrent AmpliSeq RNA plugin using the unique mapping option.

### CD11c-DTR Bone Marrow Chimeras and CD11c Depletion

To generate CD11c-DTR bone marrow chimeras 8-week-old NOD mice were lethally irradiated with two doses of 500 Rads. 10^7^ CD11c-DTR bone marrow cells were transferred i.v. after irradiation. Mice were allowed to reconstitute their hematopoietic cells for >10 weeks. In order to deplete CD11c^+^ cells in the islets, two 200 ng doses of diphtheria toxin (Sigma) were administered i.p. 24 h apart. By 24 h after the second dose, >90% of CD11c^+^ cells were depleted. If there was not >90% CD11c depletion compared to the average of the age matched controls for each experiment, these mice were excluded from analysis due to incomplete depletion.

### Flow Cytometry Analysis

To analyze trafficking of dye-labeled lymphocytes, tissues were digested and stained with the following antibodies: CD45 BUV395 (BD), CD45 Pacblue (Biolegend), CD11c FITC (Biolegend), CD4 BV711 (Biolegend), CD8 PE (eBioscience), CD8 PE-Cy7 (eBioscience), and CD19 BV510 (Biolegend). For intracellular chemokine staining of CXCL16 (R&D Systems) was performed using the FoxP3 intracellular staining kit (eBioscience); mice were treated i.v. with Berfeldin A (Sigma) for 4 h prior to harvest. For vascular adhesion molecule and chemokine expression a combination of the previous antibodies were used as well as CD31 PE (eBioscience), CD54 FITC (eBioscience), and CD106 PE (eBioscience). All antibody staining was done for 30 min on ice. Samples were collected on either a BD LSRII or LSR Fortessa and analyzed by FlowJo.

### 2-Photon Imaging of Islets

Islets were imaged using an Olympus FV100MPE (7, 8). Excitation was 810 nm and emission was detected in four channels: 450–490 nm, 500–550 nm, 575–640 nm, and 645–685 nm. Islets were scanned with 3 μm spacing in the z plane with 509 μm^2^ xy planes and a resolution of 0.994 μm/pixel. Analysis and quantitation of imaging data was done on Imaris (Bitplane).

#### Whole Stained Islets

Activated BDC-2.5 T cells were transferred 2 h prior to harvest. Whole islets were stained with 2–3 μg of antibody for CD31 and CD90.2 for 45 min on ice and then fixed with 1% PFA (Sigma) as previously described (15).

#### Intravital Islet Imaging

Intravital imaging was done as previously described (8). Briefly, mice were anesthetized with ketamine/xylazine and maintained using inhaled isoflourane (2–3% in O_2_) on a heat pad to maintain 37°C body temperature. To label the vasculature, 70 kDa dextran-FITC (Invitrogen) was injected i.v. Activated BDC-2.5 T cells were transferred 24 h prior to imaging, for analysis of islet infiltration, and 30 min prior to imaging for analysis of T cell extravasation. The pancreas was surgically exposed and islets were imaged for 30 min segments, up to 2 h, through a heated suction window to maintain the pancreas at 37°C.

### Statistical Analyses

Graphing and statistical analysis of all flow cytometry and microscopy data was performed using Prism6 (Graphpad). Statistical analyses and normalization of the RNA-seq transcriptome data were performed in R statistical language (84). Gene counts were normalized with *DEseq2* (85). Gene expression plots were created using the *heatmap3* package (86).

## Author Contributions

AS, RL, MS, JJ, and RF provided intellectual input and designed the experiments. AS, RL, and JW preformed experimental procedures. AS and RL performed data analysis. CR and MS performed RNAseq. ND and MS performed RNAseq analysis. BB and KH provided experimental mice. DS, AG, and Y-GC generated and provided NOD.CXCR6^−/−^ mice. AS, RL, ND, and RF made significant contributions to writing the manuscript. RF acquired funding and supervised the project.

### Conflict of Interest Statement

The authors declare that the research was conducted in the absence of any commercial or financial relationships that could be construed as a potential conflict of interest.

## Supplementary Material

Figure S1 BDC-2.5 T cells are largely located in extravascular regions of the islet at 24 h post-transfer. Experimental setup as described for Figure 1. Islet antigen-specific BDC-2.5 T cells were antigen-activated, fluorescently labeled, and transferred. Twenty-four hours post-transfer, islets were imaged intravitally by 2-photon microscopy. **(A)** Representative islet image (dashed line) with transferred T cells (blue), and vascular volume (red). Intravascular T cells are highlighted with yellow spheres. **(B)** Percentage of extravascular T cells within individual islets. *n* = 17 islets from 9 experiments.

Figure S2 Numbers of intravascular T cells that adhere, release, and extravasate within infiltrated islets. **(A)** Absolute numbers of cells within islets represented in Figure 1C. *n* = 6 islets from 5 mice in 5 experiments. Error bars = SEM. ^*^*P* < 0.05 calculated by Students *T*-test.

Figure S3 NOD.CXCR6^−/−^ mice lack the CXCR6 gene and protein expression, but do not have altered T1D disease progression. **(A,B)** Confirmation of CRISPR mediated CXCR6 gene knock out. **(A)** PCR confirmation that the 7 base pair deletion in NOD.CXCR6^−/−^ prevents binding of the forward primer and amplification of 280 base pair CXCR6 gene fragment. **(B)** Wild type and CXCR6^−/−^ T cells were activated by plate-bound αCD3 and soluble αCD28 antibodies. After 6 days, CXCR6 protein expression on T cells was analyzed by flow cytometry. **(C)** T1D disease progression of NOD.CXCR6^−/−^ compared to WT NOD mice in our colony. Diabetes was defined as two consecutive weeks with blood glucose readings above 300 mg/dl. No significance by Log-rank test.

Figure S4 Islet trafficking is not impaired in CXCR3^−/−^ CXCR6^−/−^ deficient T cells. Infiltration of the islets was induced by transferring OT-I CD8 T cells into C65BL/6.RIP-mOVA mice. WT and CXCR3^−/−^ CXCR6^−/−^ T cells were activated by αCD3 and αCD28, differentially fluorescent dye-labeled, and co-transferred 6 days after OT-I transfer. 24 h later islets were isolated, digested, and the transferred cells were quantified by flow cytometry. **(A)** Schematic of experimental setup. **(B)** Representative flow plots of CD45+ cells comparing trafficking of WT and CXCR3^−/−^ CXCR6^−/−^ T cells to previously infiltrated islets. Red numbers represent the number of cells in the adjacent gate. **(C)** Ratio of transferred CXCR3^−/−^ CXCR6^−/−^ to WT T cells in each tissue analyzed. Statistics: One sample *T*-test with hypothetical value = 1. **(D,E)** Number of WT and CXCR3^−/−^ CXCR6^−/−^ T cells in **(D)** islets **(E)** ILN, normalized to the number of islets isolated. Error bars = SEM. Statistics: Students *T*-test. **(C–E)** *n* = 6 mice from 3 experiments.

Video 1 T cell extravasation into the islets is an extended process. Video of Figure 1B. Extravasation of transferred BDC-2.5 T cells (green) from islet vasculature (red). Two transferred T cells undergoing extravasation from islet vasculature imaged intravitally by 2-photon microscopy. White track of motion signifies movement of intravascular T cell and the track turns blue when the T cell completes extravasation. Time stamp = min:sec; Scale bar = 10 μm.

Video 2 T cells arrest in close proximity to CD11c^+^ cells in the islet vasculature. Video of Figure 2C. 3-Dimensional rendering of the fluorescence in the boxed region in Figure 2A. Arrested T cell (blue) in contact with CD11c cell (green) through islet vasculature (red). Time stamp = min:sec; Scale bar = 10 μm.
