# Supplementary figures and images for: CD11c+ Cells Are Gatekeepers for Lymphocyte Trafficking to Infiltrated Islets During Type 1 Diabetes
Source: Front Immunol. 2019 Jan 31;10:99. doi: 10.3389/fimmu.2019.00099 (PMC6365440; doi:10.3389/fimmu.2019.00099)

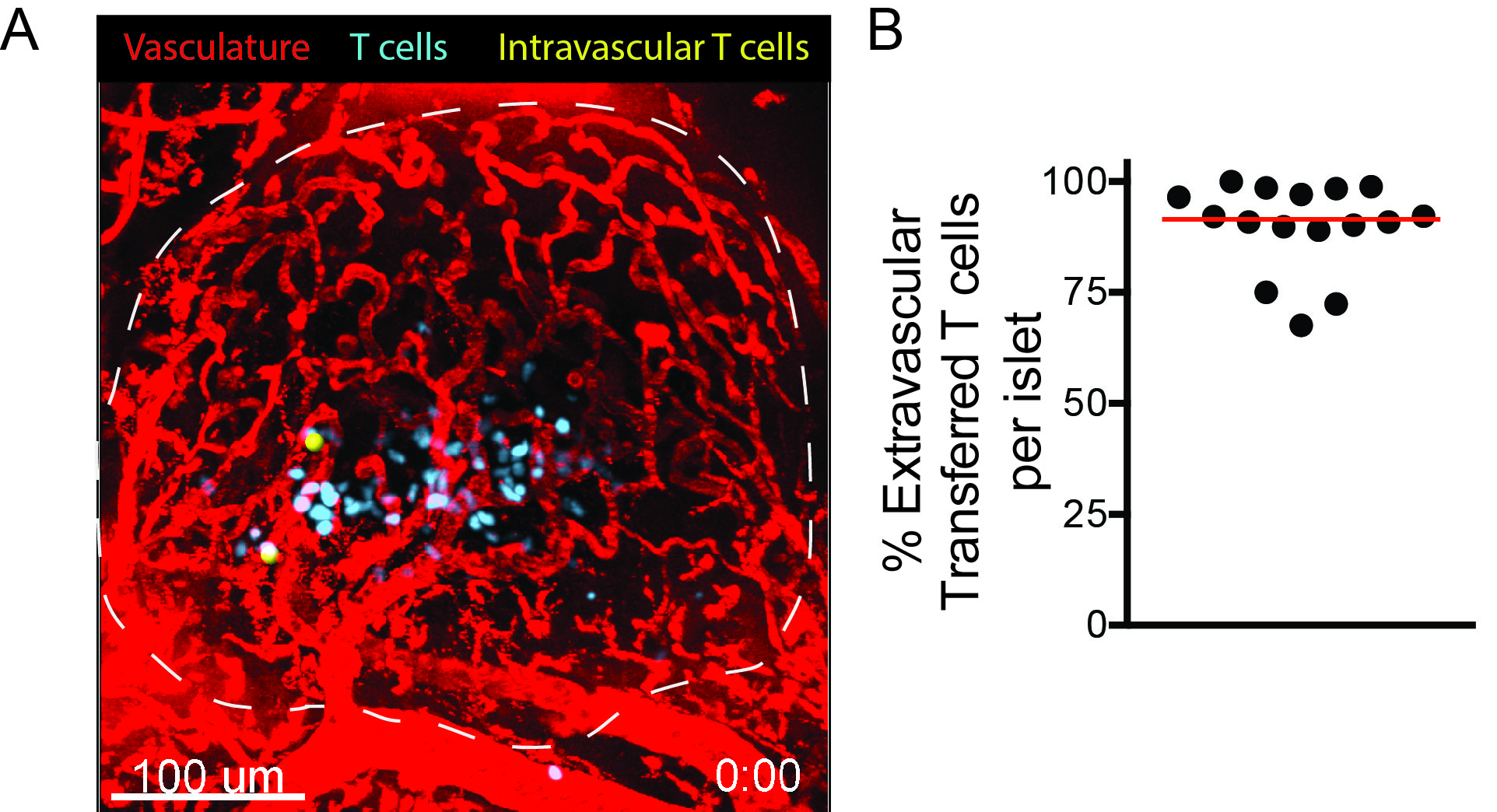

Supplement: Figure S1 — BDC-2.5 T cells are largely located in extravascular regions of the islet at 24 h post-transfer. Experimental setup as described for Figure 1. Islet antigen-specific BDC-2.5 T cells were antigen-activated, fluorescently labeled, and transferred. Twenty-four hours post-transfer, islets were imaged intravitally by 2-photon microscopy. (A) Representative islet image (dashed line) with transferred T cells (blue), and vascular volume (red). Intravascular T cells are highlighted with yellow spheres. (B) Percentage of extravascular T cells within individual islets. n = 17 islets from 9 experiments. [file Image_1.JPEG]

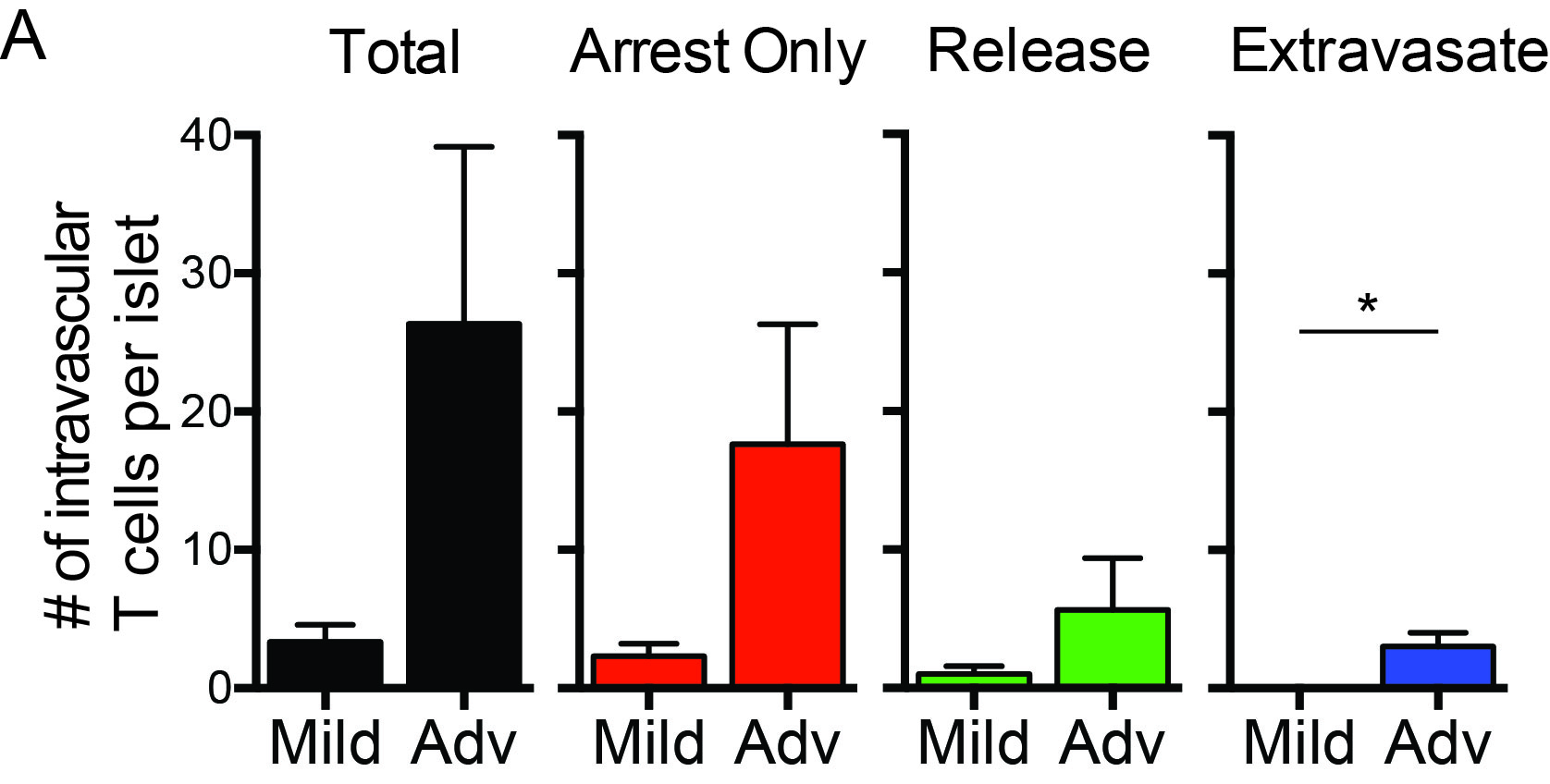

Supplement: Figure S2 — Numbers of intravascular T cells that adhere, release, and extravasate within infiltrated islets. (A) Absolute numbers of cells within islets represented in Figure 1C. n = 6 islets from 5 mice in 5 experiments. Error bars = SEM. *P < 0.05 calculated by Students T-test. [file Image_2.JPEG]

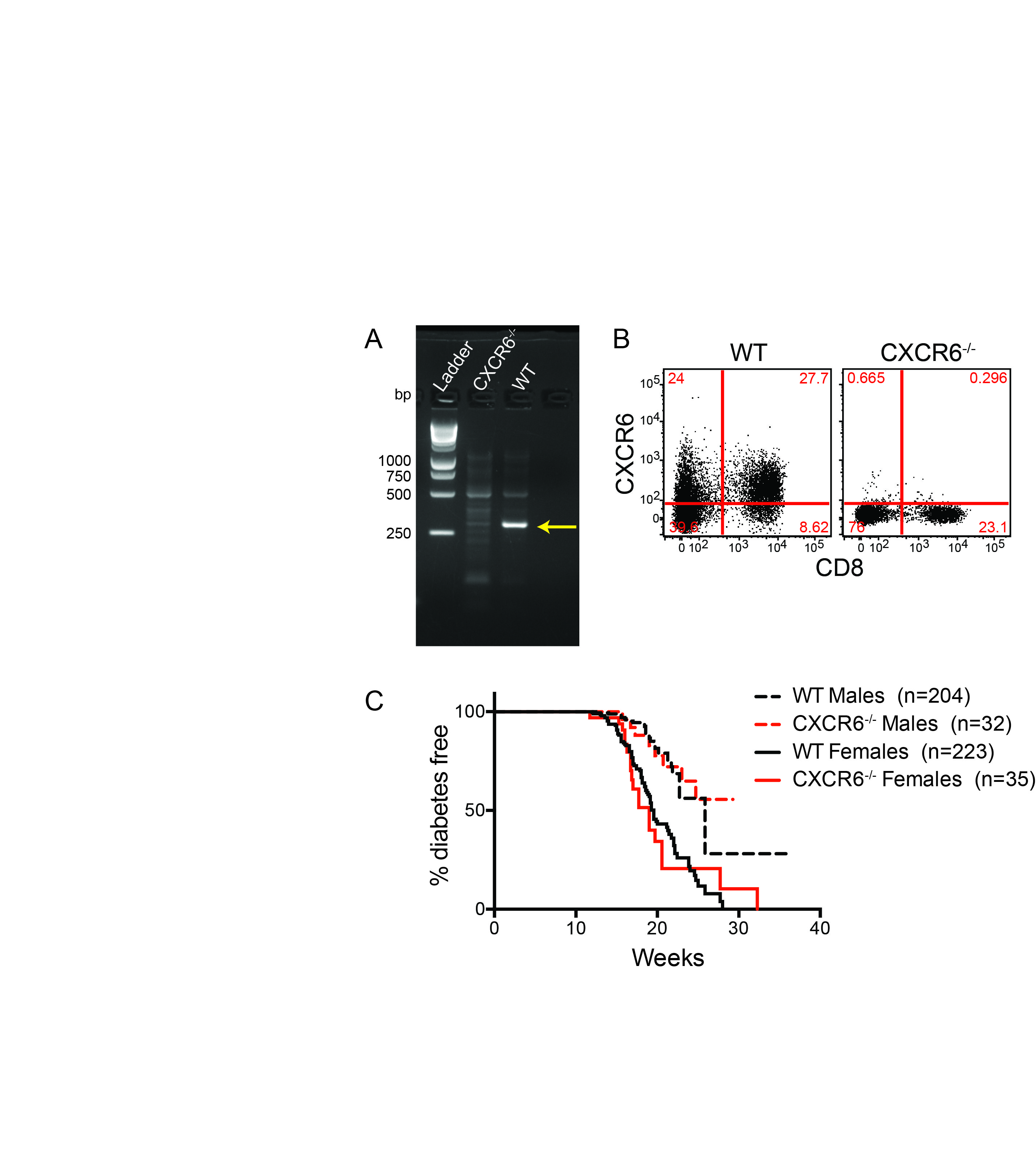

Supplement: Figure S3 — NOD.CXCR6−/− mice lack the CXCR6 gene and protein expression, but do not have altered T1D disease progression. (A,B) Confirmation of CRISPR mediated CXCR6 gene knock out. (A) PCR confirmation that the 7 base pair deletion in NOD.CXCR6−/− prevents binding of the forward primer and amplification of 280 base pair CXCR6 gene fragment. (B) Wild type and CXCR6−/− T cells were activated by plate-bound αCD3 and soluble αCD28 antibodies. After 6 days, CXCR6 protein expression on T cells was analyzed by flow cytometry. (C) T1D disease progression of NOD.CXCR6−/− compared to WT NOD mice in our colony. Diabetes was defined as two consecutive weeks with blood glucose readings above 300 mg/dl. No significance by Log-rank test. [file Image_3.jpeg]

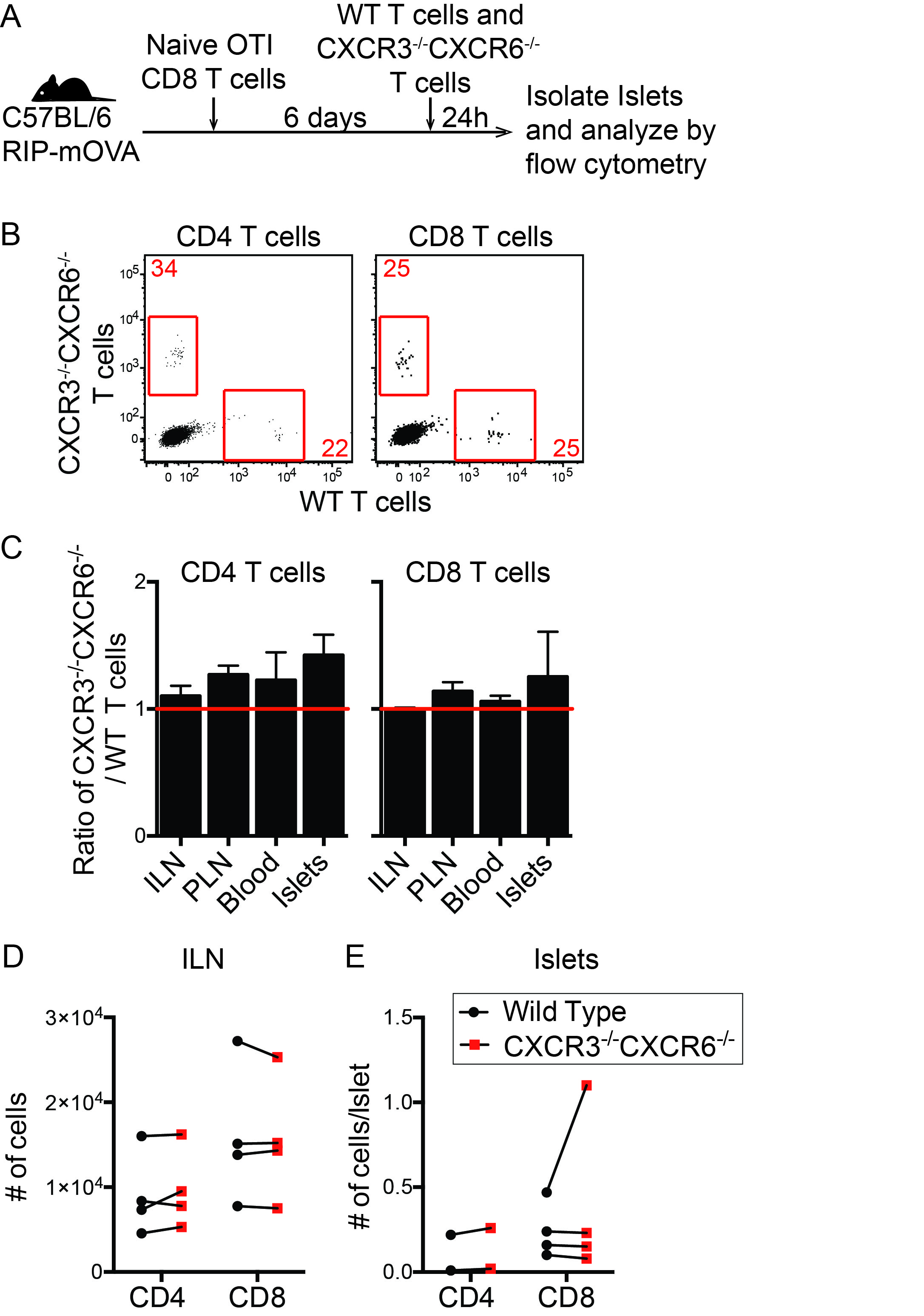

Supplement: Figure S4 — Islet trafficking is not impaired in CXCR3−/− CXCR6−/− deficient T cells. Infiltration of the islets was induced by transferring OT-I CD8 T cells into C65BL/6.RIP-mOVA mice. WT and CXCR3−/− CXCR6−/− T cells were activated by αCD3 and αCD28, differentially fluorescent dye-labeled, and co-transferred 6 days after OT-I transfer. 24 h later islets were isolated, digested, and the transferred cells were quantified by flow cytometry. (A) Schematic of experimental setup. (B) Representative flow plots of CD45+ cells comparing trafficking of WT and CXCR3−/− CXCR6−/− T cells to previously infiltrated islets. Red numbers represent the number of cells in the adjacent gate. (C) Ratio of transferred CXCR3−/− CXCR6−/− to WT T cells in each tissue analyzed. Statistics: One sample T-test with hypothetical value = 1. (D,E) Number of WT and CXCR3−/− CXCR6−/− T cells in (D) islets (E) ILN, normalized to the number of islets isolated. Error bars = SEM. Statistics: Students T-test. (C–E) n = 6 mice from 3 experiments. [file Image_4.jpeg]
